# Multi‐Scale Label‐Free Human Brain Imaging with Integrated Serial Sectioning Polarization Sensitive Optical Coherence Tomography and Two‐Photon Microscopy

**DOI:** 10.1002/advs.202303381

**Published:** 2023-10-26

**Authors:** Shuaibin Chang, Jiarui Yang, Anna Novoseltseva, Ayman Abdelhakeem, Mackenzie Hyman, Xinlei Fu, Chenglin Li, Shih‐Chi Chen, Jean C. Augustinack, Caroline Magnain, Bruce Fischl, Ann C. Mckee, David A. Boas, Ichun Anderson Chen, Hui Wang

**Affiliations:** ^1^ Department of Electrical and Computer Engineering Boston University 8 St Mary's St Boston 02215 USA; ^2^ Department of Biomedical Engineering Boston University 44 Cummington Mall Boston 02215 USA; ^3^ Department of Mechanical Engineering The Chinese University of Hong Kong Hong Kong 999077 China; ^4^ Department of Radiology Massachusetts General Hospital A.A. Martinos Center for Biomedical Imaging 13th Street Boston 02129 USA; ^5^ VA Boston Healthcare System U.S. Department of Veteran Affairs Boston 02132 USA; ^6^ Boston University Chobanian and Avedisian School of Medicine Boston University Alzheimer's Disease Research Center and CTE Center Boston 02118 USA; ^7^ Department of Neurology Boston University Chobanian and Avedisian School of Medicine Boston 02118 USA; ^8^ Department of Pathology and Laboratory Medicine Boston University Chobanian and Avedisian School of Medicine Boston 02118 USA; ^9^ VA Bedford Healthcare System U.S. Department of Veteran Affairs Bedford MA 01730‐1114 USA

**Keywords:** human brain mapping, microvasculature, multi‐contrast Imaging, myelinated fibers, neurodegeneration, neurons, Volumetric microscopy

## Abstract

The study of aging and neurodegenerative processes in the human brain requires a comprehensive understanding of cytoarchitectonic, myeloarchitectonic, and vascular structures. Recent computational advances have enabled volumetric reconstruction of the human brain using thousands of stained slices, however, tissue distortions and loss resulting from standard histological processing have hindered deformation‐free reconstruction. Here, the authors describe an integrated serial sectioning polarization‐sensitive optical coherence tomography (PSOCT) and two photon microscopy (2PM) system to provide label‐free multi‐contrast imaging of intact brain structures, including scattering, birefringence, and autofluorescence of human brain tissue. The authors demonstrate high‐throughput reconstruction of 4 × 4 × 2cm^3^ sample blocks and simple registration between PSOCT and 2PM images that enable comprehensive analysis of myelin content, vascular structure, and cellular information. The high‐resolution 2PM images provide microscopic validation and enrichment of the cellular information provided by the PSOCT optical properties on the same sample, revealing the densely packed fibers, capillaries, and lipofuscin‐filled cell bodies in the cortex and white matter. It is  shown that the imaging system enables quantitative characterization of various pathological features in aging process, including myelin degradation, lipofuscin accumulation, and microvascular changes, which opens up numerous opportunities in the study of neurodegenerative diseases in the future.

## Graphical abstract

The integrated serial sectioning polarization sensitive optical coherence tomography (PSOCT) and two‐photon microscopy (2PM) system provides label‐free imaging of birefringence (|*n_e_* − *n_o_* |), scattering (µ_*s*_), and autofluorescence of human brain tissue that allows for quantitative measurement of myelin content, vascular structure, and cellular information that can be applied in the study of brain aging and neurodegeneration.

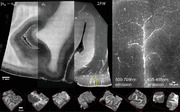

## 1 Introduction

The human brain is organized at multiple scales, from the macroscopic white‐grey matter structure to the microscopic components of neurons, axons, vascular structures. Mapping brain structures at different scales is essential for functional and pathological studies. Magnetic resonance imaging (MRI) is widely used for mapping the brain's macroscopic structure,^[^ ^1^, ^2^, ^3^ ^]^ however, it is limited in its ability to achieve the microscopic spatial resolution. Label‐free, automatic serial sectioning optical coherence tomography (OCT) is complementary to traditional histology as it generates depth‐resolved 3D images and allows intact tissue structure to be reconstructed. It also utilizes intrinsic scattering contrasts to measure tissue structure and composition and has demonstrated utility in mouse and human brain.^[^ ^4^, ^5^, ^6^ ^]^ Polarization‐sensitive OCT (PSOCT) measures the birefringence properties of myelinated fiber tracts to provide additional contrast.^[^ ^7^, ^8^, ^9^ ^]^ Wang et al^[^ ^10^ ^]^ demonstrated a fully automated pipeline using a serial sectioning PSOCT to image tens of cubic centimeters of brain tissue, enabling quantitative estimation of cortical layer thickness and generation of 2D microscopic images that were useful for orientation in tractography. While PSOCT lacks the resolution and sensitivity to visualize individual neurons, axons, and capillaries, optical coherence microscopy (OCM) is capable of visualizing neurons in human^[^ ^11^ ^]^ and mouse brain.^[^ ^12^ ^]^ Drawbacks of OCT/OCM include the requirement for depth scanning, which slows imaging of large tissue volumes, and speckle noise, that interferes with accurate neuronal counts.^[^ ^11^, ^12^ ^]^

Two‐photon microscopy (2PM) is an optical imaging technique that suppresses out‐of‐focus fluorescence and generate high‐resolution images with an excellent signal‐to‐background ratio. This technique has been applied to a variety of tissue types, including skin,^[^ ^13^, ^14^ ^]^ retinal,^[^ ^15^, ^16^ ^]^ cancerous tissue,^[^ ^17^, ^18^ ^]^ and brain.^[^ ^19^, ^20^ ^]^ Multiple endogenous fluorophores are two‐photon excitable, such as collagen and elastin fibers,^[^ ^21^ ^]^ iron‐free variants of hemoglobin and macromolecules in blood plasma,^[^ ^22^ ^]^ and lipofuscin^[^ ^23^ ^]^ in aging neurons. The presence of these endogenous fluorophores in the postmortem human brain offers opportunities for label‐free, high‐resolution imaging of neurons, capillaries, and axonal fibers, which can complement the images provided by PSOCT.

Here, we present our efforts to combine serial sectioning PSOCT and 2PM to image large‐volume human brain samples at multiple scales. Our results show that by integrating the scattering, birefringence, and autofluorescence contrasts, we were able to investigate multiple perspectives of myelin content, vascular structure, and cellular structures. Previously, hybrid systems combining OCT and 2PM were applied in wound healing,^[^ ^24^ ^]^ microvascular‐flow distribution,^[^ ^25^ ^]^ skin inflammation^[^ ^14^ ^]^ and skin diseases.^[^ ^18^ ^]^ To our knowledge, no previous work has combined serial sectioning, PSOCT, and 2PM in imaging human brain tissue. Our system is capable of simultaneously imaging and registering key features in human brain, opening a window for investigating their spatial interactions and pathological relevance in brain aging and neurodegeneration.

## 2 Results

### 2.1 Serial Sectioning PSOCT‐2PM Microscope

**Figure 1:**
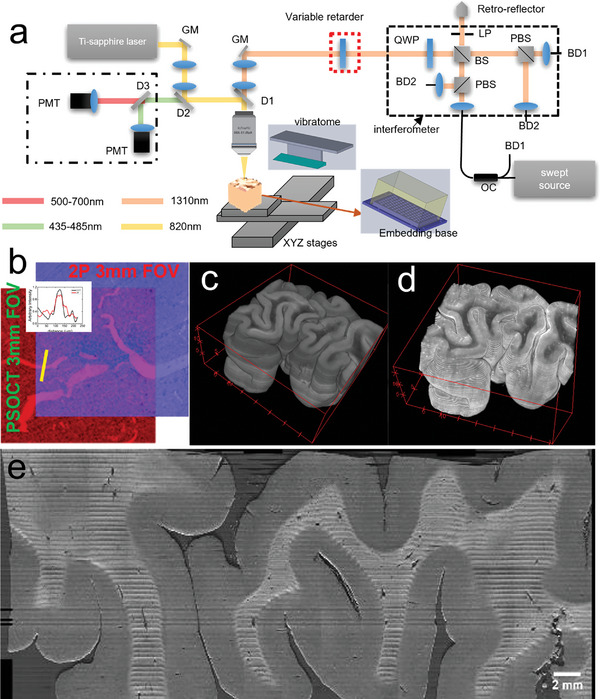
PSOCT‐2PM system schematic and volumetric reconstructions from a 4 x 4 x 2 cm^3^ human brain sample. a) Optical layout of the hybrid system. PMT: photomultiplier tube. D1‐D3: Dichroic mirrors. GM: Galvo mirror pair. QWP: quarter wave plate. LP: linear polarizer. PBS: polarized beam splitter. BS: beam splitter. BD: balanced detectors. OC: optical circulator. The sample embedding is shown in the inset. A 3D printed sample plate was used to hold the whole block and screwed to the bath. b) Illustration of the overlapping 2PM and PSOCT FOVs. Inset shows the line profile of a vessel in the overlapping area. (c‐d) Volumetric rendering of PSOCT c) and 2PM d) images of a human brain sample. Both volumes were down‐sampled to an isotropic voxel size of 60 µm for easier rendering, XYZ axis units are in mm. e) XZ plane of PSOCT volume shows the continuous axial structure.

The serial sectioning PSOCT‐2PM system setup is shown in **Figure** **1**a and a detailed description is included in the Methods section. The excitation wavelength of 2PM was 820 nm and two detection channels were used: one covering 460 ±  25 nm (short wavelength channel) for detecting autofluorescence from elastin, collagen, blood, and fixation agent, and the other covering 600  ±  100 nm (long wavelength channel) for detecting autofluorescence from lipofuscin. Using a 4x objective lens, the lateral resolution is 2.4 µm and the axial resolution (depth of focus) is 48 µm (shown in Figure S1, Supporting Information). Depending on the features of interest, two sets of imaging configurations were employed, 1) 3 × 3 mm^2^ Field of View (FOV) with 2 µm pixel step size for imaging large samples, 2) 1.5 × 1.5mm^2^ FOV with 1 µm pixel step size for optimizing capillary signals. The PSOCT setup is shown on the right part of Figure 1a. Its FOV was kept the same as the 2PM, with a 3 µm pixel step size yielding a 6 µm isotropic resolution and 150 µm depth of focus (shown in Figures S1, and S2, Supporting Information). Under the objective, the sample was embedded in 4% agarose and mounted on XYZ motorized stages using a 3D printed base plate (shown in inset) to secure it for measurements spanning multiple days. The stages translated the sample under the objective to cover the whole area, as well as between the vibratome and the objective. To obtain large volumetric imaging, a customized vibratome^[^ ^26^ ^]^ equipped with a 2.5‐inch wide sapphire blade was integrated into the imaging system. The vibratome was designed to minimize out‐of‐plane vibration for optimal cutting performance.^[^ ^27^, ^28^ ^]^ The cutting thickness can be set from 30 to 450 µm, depending on the effective imaging depth in the sample and the requirements for the subsequent histology.

We employ a few distortion correction steps as described in the methods session to ensure the stitched image volumes free of distortion. Since PSOCT and 2PM used the same objective lens albeit different optical paths before the objective, a linear translation of between their FOVs was effective for cross‐modality registration. Figure 1b shows the registration of the two FOVs for a region with multiple vessels. The co‐aligned vessels in the overlap area and their line profiles in the inset show the successful registration between the two modalities. Figure 1c‐d shows the volumetric reconstruction of the PSOCT and 2PM images of a 4×4×2cm^3^ sample from Broca's area 44/45 (BA44/45). The sample was index‐matched to reduce the scattering and increase the penetration depth of both PSOCT and 2PM. In PSOCT volume (c) white matter appeared as darker than the grey matter due to a higher scattering coefficient. 2PM volume (d) that were registered to PSOCT also presented a contrast between grey and white matter. The autofluorescence signals were stronger in grey matter than white matter, which may be due to the higher concentration of autofluorescent components such as elastin‐rich extra cellular matrix^[^ ^29^ ^]^ and vessels, as well as lipofuscin‐filled neurons.^[^ ^23^ ^]^ Figure 1e shows an XZ plane of the PSOCT volume, demonstrating the continuous structure along the axial direction, including the intact cortex surface and continuous vessel cross‐sections. A volumetric fly‐through of the PSOCT and 2PM images is included in the supplementary Videos S1, and S2, Supporting Information.

**Figure 2:**
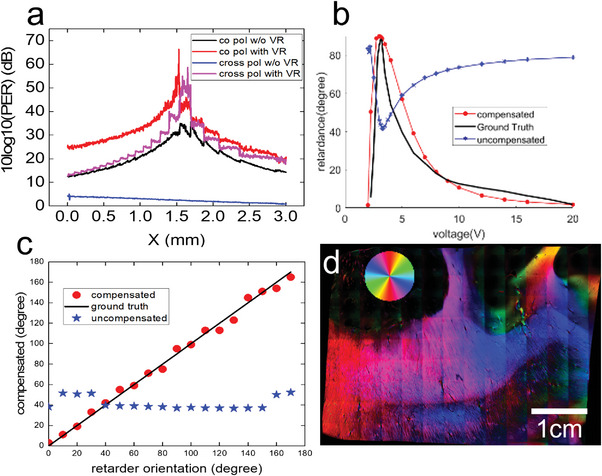
Characterization of polarization extinction ratio (PER), retardance, and orientation before and after birefringence compensation using an electrically modulated variable retarder. a) PER along the *x*‐axis at the center of FOV for cross and co polarization channels before and after compensation of birefringence. b) Retardance measurement before (blue) and after (red) birefringence compensation using a variable retarder as a sample, compared with ground truth (black) of the retardance provided by manufacturer. c) Comparison of orientation measurement of a retarder as a sample, before (blue) and after (red) birefringence compensation. The black line indicates the physical orientation of the retarder. d) Retardance weighted fiber optic orientation map of a brain sample with index matching. The color wheel indicates the axonal fiber orientation in the imaging plane, which is perpendicular to the optic‐fast axis of the local birefringence.

Birefringence of brain tissue is an important feature exploited by PSOCT to provide contrast that is sensitive to myelin content and axonal orientation. However, when combining PSOCT with 2PM, the optics added to the PSOCT light path such as the dichroic mirror and the telescope introduced a system birefringence, preventing an accurate measure of sample birefringence. To compensate this system birefringence, we used an electrically modulated variable retarder (Thorlabs Inc, red dashed box in Figure 1a) that was oriented with the same optic axis but opposite retardance as the optics in the sample arm. **Figure** **2** shows a comparison of the polarization extinction ratio (PER), dynamic range of retardance, and optic axis orientation before and after system birefringence compensation. Before compensation, the PER (Figure 2a) of co‐polarization channels was below 20 dB off the center of the FOV, and the PER of cross‐polarization channels was below 5 dB. After compensation, PER increased by 10–30 dB, recovering PSOCT's ability to measure sample birefringence. Figure 2b shows the retardance measurement before and after compensation. Before compensation, the hybrid system produced a background retardance of ≈70° and the measurement on the sample was inaccurate. After compensation, the background was removed and the dynamic range of retardance measurement was recovered to ≈90°. The measurement matched to the ground truth of retardance provided by the manufacturer (black line). Figure 2c shows the orientation measurement before and after compensation. Before compensation, the measurement could not reveal different orientations set on the retarder at all. After compensation, the measured orientation matched the physical orientation of the retarder and the dynamic range was recovered to 180°. Figure 2d shows the retardance weighted orientation image of a 4×4cm^2^ brain sample after birefringence compensation. The sample was index‐matched to increase the signal‐to‐noise ratio (SNR), which also increased the accuracy of orientation measurement.^[^ ^30^ ^]^ The color‐coded fiber orientation across this brain region was clear and matched with the imaging features revealing the fiber structures.

### 2.2 Myelin Content

**Figure 3:**
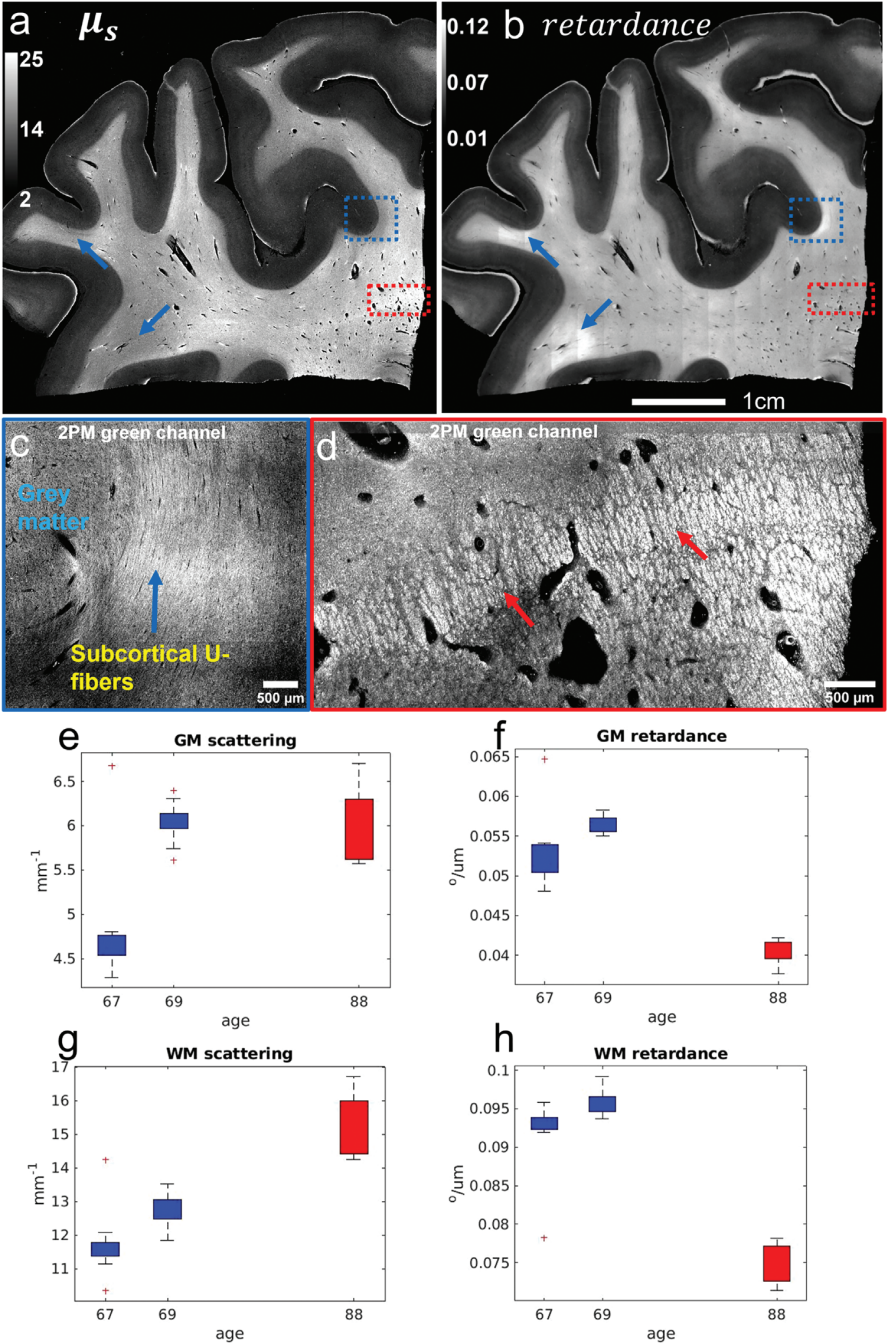
Comparison of different optical properties from PSOCT and the corresponding structure in 2PM. a,b) Estimated µ_*s*_ (a) and retardance (b) from the same slice of a brain sample block. c,d) zoom‐in of the blue and red ROIs by 2PM autofluorescence. (c) The blue arrows point to the parallelly oriented U‐fibers underneath the cortex, which show high signals in the retardance map. (d) Red arrows point to the cross sections of densely packed myelinated fibers, corresponding to the increased scattering in the µ_*s*_ map. e–h) Quantitative comparison of µ_*s*_ and retardance for two samples of late 60s and one sample of 88‐year old. White and gray matter were analyzed separately. The box plot was generated from 10 slices from each sample, with each slice providing a mean value of µ_*s*_ and retardance. The red + indicates outliers in the box plot analysis.

Optical properties estimated from PSOCT allow for the quantification of myelin content from different perspectives. Scattering coefficient (µ_*s*_)has been related to the myelin density across different human brain regions.^[^ ^31^ ^]^ The retardance of brain tissue has been found to be related to myelin integrity and fiber orientation.^[^ ^32^ ^]^ **Figure** **3**a,b shows the retardance and µ_*s*_ for the same slice. While they share similar white‐gray matter contrast, there are local differences, such as in the branches of white matter (blue arrows), the sub‐cortical U‐fibers (blue ROI), and a deep white matter region (red ROI). We use 2PM to elucidate the microstructure of fibers at those regions, which has better resolution and is speckle‐free. The sub‐cortical u‐fibers show a strong autofluorescence with 2PM (c). Since the u‐fibers are highly parallel (yellow arrows), accumulation of birefringence from each individual fiber results in the enhanced signal in the retardance map. The deep white matter region also reveals strong autofluorescence, which manifests as cross‐sections of large fiber bundles with an oblique angle (d). Considering the strong scattering of densely packed fibers, these large fiber bundle structures may be the microscopic origin of the increased µ_*s*_. In short, our hybrid system allows for the mesoscopic measurement of myelin content from scattering and birefringence, and 2PM provides a microscopic validation. Based on the quantitative measurement of myelin content using these different optical properties, we demonstrate the ability to study the demyelination process in aging. Figure 3.e‐h shows the comparison of scattering and retardance of two late 60 years old samples and one late 80 years old samples that are neurologically normal. Two more normal control samples were excluded due to having more than 24 hours of Post Mortem Interval (PMI), comparison of all five samples can be found in Figure S7, Supporting Information. In gray matter, the scattering difference is not apparent, but the retardance was found to decrease with inceasing age. In the white matter, the young age group has a lower scattering and higher retardance comparing to older age group. While this is a small demonstrate with only three samples, it illustrates our ability to reveal differences in myelin characteristics with aging.

### 2.3 Vascular Structure

**Figure 4:**
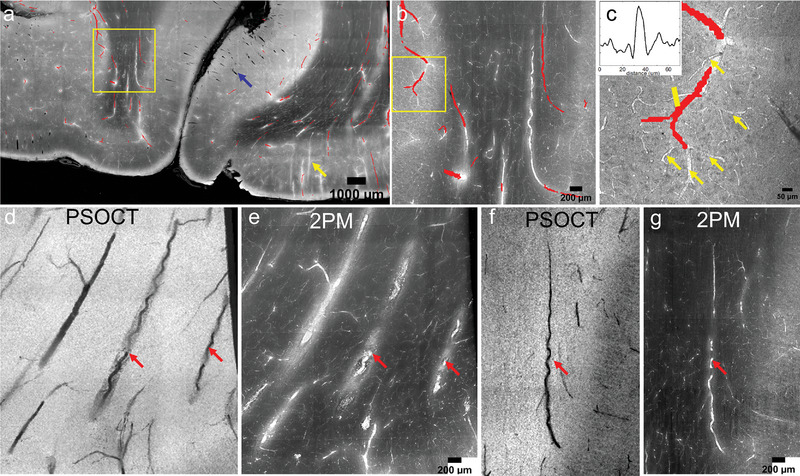
Vascular image from PSOCT and 2PM. First row, registered OCT vessel segmentation (red) with 2PM images at three different scales. a) Blue arrow: dark structures show the empty space within vessel. Yellow arrow: bright vessels due to residual blood within the vessel. b) Zoom in of the small vessels and capillaries in white and gray matter. c) Yellow arrows: extension of vessels from the large vessel visible in OCT. Inset shows the good SNR of a 10 µm diameter vessel. d‐g) examples of spiraling vessels that are more clear and continuous in OCT than in 2PM.

OCT has proven to be effective for imaging vascular structure of the human brain. Previous studies have shown quantitative analysis of the vascular geometry using graph theory following vessel segmentation.^[^ ^5^, ^33^ ^]^ However, due to the resolution limit and speckle noise, it has been challenging for OCT to visualize capillaries and small vessels less than 20 µm in diameter, which we can be complemented by using 2PM. Here we use the hybrid system to elucidate the vascular structure in an early‐stage Chronic Traumatic Ecephalopathy (CTE) sample, which unveiled a mild modification in vessel morphology and perivascular space. **Figure** **4**a–c overlay the co‐registered PSOCT vessel segmentation (red segments) on 2PM images. The PSOCT vessels were segmented from volumetric µ_*s*_ image of 150 µ*m* thick, while the 2PM image is the max intensity projection of three consecutive frames, each covering a depth of 48 µm, which combined covers the same depth range as PSOCT. The PSOCT segmentation identifies the large vessels, while 2PM clearly reveals many more small vessels. Some of 2PM vessels are bright (yellow arrow), due to residual blood within the vessel, and others are dark (blue arrow), revealing the empty space within the vessel. The zoom‐in image in (b) shows the small vessel and capillaries in both white and gray matter that are challenging to resolve from PSOCT. (c) shows an example where 2PM extends the PSOCT vessel segmentation by revealing the small vessels branching from the large vessel (yellow arrows). The inset shows we have good signal‐to‐noise ratio of vessels that are 10µ*m* in diameter.

While 2PM is excellent at revealing large and small vessels, due to the limited depth of focus, it may have trouble tracing large vessel that are longer than the depth of focus. One such example is the spiraling vessels revealing altered morphology. The second row of Figure 4 shows a few comparisons of spiraling vessels from an PSOCT minimum intensity projection and 2PM max intensity projection (since they appear bright in 2PM). The spiraling vessels are continuous on PSOCT, and the spiraling shape is clearer, from which calculating vessel morphologies such as tortuosity can be accurately performed. In contrast, 2PM only shows segments of the spiraling vessels intermittently because of our 2PM scanning procedure. Interestingly, 2PM also reveals high density spots surrounding the spiral vessels which indicates a modified perivascular space (e). In general, 2PM complements PSOCT with small vessels and capillaries, while the 3D imaging ability of PSOCT is useful when quantifying more mesoscopic structures such as long spiraling vessels.

### 2.4 Lipofuscin and Neurons

**Figure 5:**
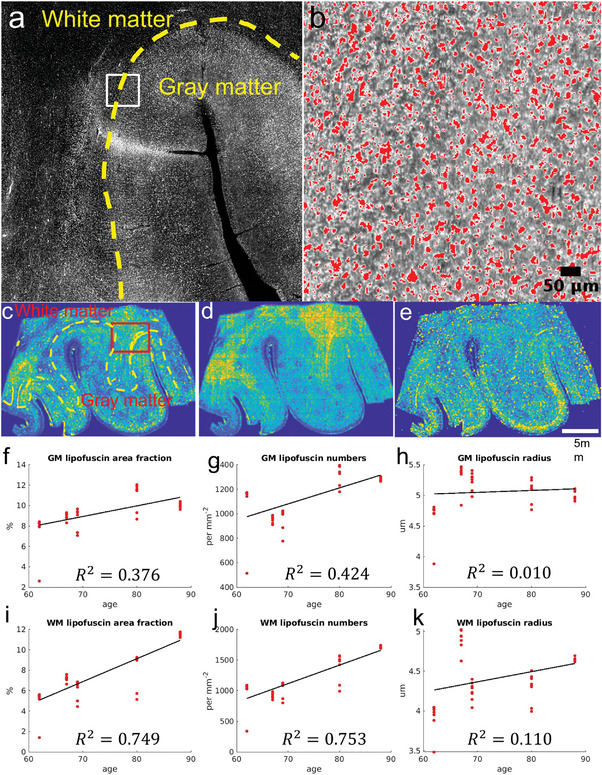
Segmentation of individual lipofuscin particles and quantitative analysis. a) 2PM image of lipofuscin particles around a sulcus in (c). b) Zoom in of the white ROI in (a) shows the quality of segmentation, red: identified lipofuscin. c–e) Quantitative metrics of lipofuscin including area fraction (%), number of lipofuscin per mm^2^, and radius (µm). f–k) Linear regression of lipofuscin metrics with age for gray matter and white matter separately. The R^2^ of least square fitting is reported for each Figure.

Lipofuscin has been regarded as an aging pigment,^[^ ^23^, ^34^, ^35^, ^36^, ^37^ ^]^ but its role in neurodegeneration has gained increasing attention recently.^[^ ^38^, ^39^, ^40^ ^]^ Our system utilized autofluorescence from the long wavelength channel of 2PM to provide a scalable mapping of lipofuscin across large samples. To confirm what we saw was lipofuscin, we used fluorescence lifetime imaging (FLIM) to validate the source of autofluorescence. We included a FLIM image in Figure S5, Supporting Information showing the ≈400 ps fluorescence lifetime of these autofluorescent structures, which agrees with a previous report of fluorescence lifetime of one major lipofuscin type around 390ps.^[^ ^41^ ^]^ We also used a lipofuscin quencher (TrueBlack, Biotium), which quenched all of the lipofuscin‐like particles, and only left Amyloid Beta‐alike particles (Figure S6, Supporting Information). We segmented lipofuscin granules and quantified the lipofuscin distribution (**Figure** **5**a,b). Lipofuscin was densely scattered in all areas including white and gray matter. The zoom‐in ROI (b) shows that most lipofuscin granules are segmented. Based on the lipofuscin segmentation, we calculated three quantitative metrics of lipofuscin distribution, including the area fraction of lipofuscin (c, unit: %), the number density map (d), and the mean radius of lipofuscin (e, unit: µm).

We investigated the change of the three lipofuscin metrics above in the white and gray matter during aging process. We analyzed the 2PM images obtained from the same samples as in Figure 3e–h. Here we include two more samples than in Figure 3e‐h as we did not find evidence that PMI affects the lipofuscin appearance. Figure 5f–k shows the linear fitting of area fraction, number density, and mean radius of lipofuscin with respect to age. In terms of the area fraction, we found that both white and gray matter have increasing lipofuscin with aging. The white matter starts with a lower lipofuscin area fraction at younger age but increases faster than gray matter. By the age of late 80s, we found that gray and white matter have about the same level of lipofuscin area fraction. For the number density, both white and gray matter increase with age. They start at about the same number at the age of early 60s and the number in white matter grows faster than gray matter with age. For the lipofuscin size, in gray matter the size remained relatively unchanged, but in white matter lipofuscin grows larger with aging. These quantitative results indicate that the lipofuscin has a greater accumulation rate in the white matter compared to the cortex at the age of 60s to 80s. The outlier of size in (k) at 67 years old was biased by the appearance of autofluorescence around the bright vessels.

**Figure 6:**
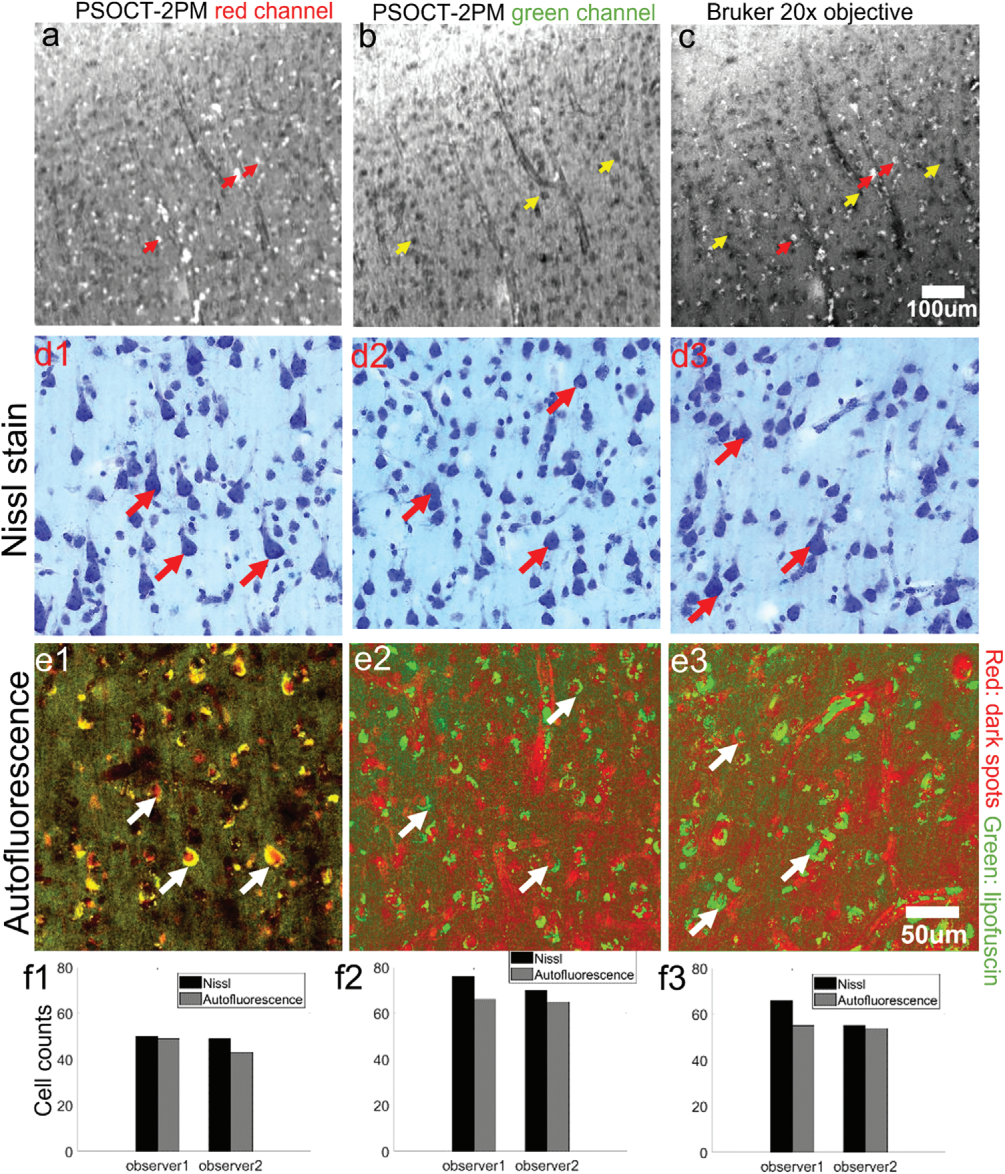
Autofluorescence images of brain tissue at different resolutions, and comparison with Nissl stain. a) Long wavelength channel of the PSOCT‐2PM system showing the lipofuscin autofluorescence. b) Short wavelength channel of the PSOCT‐2PM system showing the dark spots. c) 0.5 µm high‐resolution image obtained with a 20x objective from the Bruker 2PM system. (d1‐d3) Three ROIs in the Nissl stain image. ROIs were taken from the grey matter. (e1‐e3) Bruker 2PM images of corresponding ROIs as in d1‐d3, with lipofuscin in the green channel and inverted dark spots in the red channel. A few representative neurons in the Nissl stain and the corresponding dark‐spot‐lipofuscin structures are highlighted (red and white arrows). (f1‐f3) Manual counts of neuron numbers from the two modalities by two observers.

Apart from being related to aging, lipofuscin may also be used as cell indicator. **Figure** **6** shows autofluorescent images from a younger age subject, there were scattered lipofuscin in the long wavelength channel (Figure 6a, red arrows) and dark spots in the short wavelength channel (Figure 6b, yellow arrows). To better visualize their location, we imaged the same region under a commercial 2PM (Bruker) at 0.5 µm resolution and found the same signals of lipofuscin and dark spots (Figure 6c, yellow and red arrows). Benefiting from the high resolution, we found that most lipofuscin was around the dark spots or the vessels. Considering that lipofuscin is accumulated in the lysosome of the cell soma, we believe this lipofuscin‐dark‐spot structure is an individual cell. To validate, we sectioned a 30 µ*m* slice after imaging with a Bruker 2PM and performed a Nissl stain on the same slice, which is a standard method for visualizing the cell soma. We selected a region enclosing layers II, III, and IV of the cortex where a high density of neurons was expected, and investigated neurons in three ROIs (Figure 6, second row: Nissl; third row: Bruker 2PM). We found the markers of lipofuscin and dark spots in the 2PM images agreed with the neurons in the Nissl stain at the same location (red and white arrows). Therefore, 2PM autofluorescence combining lipofuscin and dark spots can be used to quantitatively identify the neurons. Visualizing cells using nonlinear imaging methods has been reported before using third harmonic generation,^[^ ^42^ ^]^ here we show that two‐photon autofluorescence is another nonlinear imaging method for label‐free imaging of cells. Two observers counted the cell number in the Nissl stain and in the autofluorescence image (Figure 6, bottom row). Both observers found similar counts of neurons in the Nissl and 2P images, and the correspondence rate between the two modalities was 92% on average.

## 3 Discussion

We have developed a hybrid system that combines serial sectioning PSOCT and 2PM and generates multi‐contrast images of large volumes of human brain tissue. One striking feature of the hybrid system is that it enables 3D reconstruction and quantitative analysis of different structural components simultaneously and across scales. The 3 × 3 mm^2^ large FOV of synchronized PSOCT and 2PM significantly enhanced the throughput of volumetric imaging. The FOV and resolution can be adjusted to meet different application requirements, either optimizing imaging speed or image resolution. Additionally, PSOCT and 2PM images share a fixed spatial registration. With optical distortion correction, a linear translation is effective to co‐register the two image volumes with a micrometer‐level precision for all the samples. Using this hybrid system, we have demonstrated the collection of multiple brain contrasts including scattering, birefringence, and autofluorescence at different scales. These contrasts enrichened the measurements of the myelin content, vascular structure, lipofuscin, and cells in human brain tissues. Importantly, we have shown that the intrinsic optical properties are sensitive to detect mild modifications in vascular morphology of early CTE, as well as to quantify gradual changes of myelin and cellular contents in normal aging brains (between 60s and 80s), which manifest differential features between white matter and cortex.

Although the combination of conventional OCT and 2PM have been reported in either hybrid or separate systems to enrich the image contrasts of the individual modalities,^[^ ^18^, ^24^, ^25^ ^]^ our study for the first time integrates PSOCT and 2PM with serial blockface imaging to enable a multi‐facet analysis of the key structures in the human brain, in tens of cubic centimeter volumes. One important challenge of combining PSOCT and 2PM into a hybrid system is the system birefringence that prevents accurate measurement of sample retardance and orientation. System birefringence originates from the optical telescopes, dichroic mirrors, and scanning mirrors, and results in a substantial bias to the polarization measurements of the sample. We removed this bias by using an adjustable retarder that enabled accurate measurement of true retardance and orientation from the sample. Quantification of these multi‐structural parameters provides insights in several perspectives for the study of aging and neurodegenerative diseases.

Myelin degradation is an important pathological feature observed in aging and various neurodegenerative diseases. Previously, frontal lobe myelin integrity measured using MRI has shown declination with aging after 40 years old.^[^ ^43^ ^]^ We have demonstrated the quantitative measurement of myelin content using µ_*s*_ and retardance by PSOCT and used 2PM to validate local variations of these optical properties. Using these parameters, we found increased scattering and reduced retardance at older age, especially in white matter (Figure 3). Our interpretation of such change is that with myelin breakdown, the lipid molecules are not efficiently recycled and form myelin defects in the brain. As birefringence manifests in organized lipid structure, the breakdown of myelin reduces retardance. However, the myelin defects still scatter, and combined with effects from lipofuscin accumulation and brain atrophy, we observe that the overall tissue scattering has a moderate increase with age. In neurodegenerative diseases, demyelination has been observed in both Alzheimer's disease (AD) patients and postmortem brain samples.^[^ ^43^, ^44^, ^45^ ^]^ MRI imaging has shown that myelin breakdown occurs in white matter tracts throughout the brain, impairing long‐range connectivity between different brain regions in AD patients.^[^ ^43^ ^]^ CTE is a neurodegenerative disease associated with repeated head injuries, particularly in individuals engaged in contact sports or military service. Demyelination has been observed in CTE cases, primarily in regions affected by repeated traumatic injury.^[^ ^46^, ^47^ ^]^ One future direction of our method is to analyze the change of myelin content using the intrinsic optical properties in large volumes of AD and CTE samples, validated by sub‐micron resolution imaging.

Changes in vascular morphology have been found in post‐mortem brain tissue including increased tortuosity as early as 55 years old,^[^ ^48^, ^49^ ^]^ increased number of spiraling and looped vessels in AD,^[^ ^50^ ^]^ and decreased capillary density in older human brains.^[^ ^51^, ^52^ ^]^ A large portion of these studies were focused on 2D images of small vessels and capillaries. We have shown that by integrating 2PM and PSOCT images, we can both trace long vessel trajectories as well as inspect small vessels and capillaries branching out from the long vessels (Figure 4, first row). The complementary information provided by the two modalities allows a full inspection of vascular network from morphology to capillary density that can be useful in the study of small vessel disease.^[^ ^53^ ^]^ It is important to emphasize that PSOCT is crucial in tracing the vessel structures in 3D. One good example is the spiraling vessel that has been related to aging^[^ ^54^ ^]^ and AD.^[^ ^55^, ^56^ ^]^ We have found PSOCT better resolves the spiraling structure, especially when the vessel is running at oblique angle to the imaging plane (Figure 4, second row).

In AD, neurons undergo degeneration and death.^[^ ^57^, ^58^, ^59^, ^60^, ^61^ ^]^ This neuronal loss is a characteristic feature that contributes to the progressive decline in cognition, particularly in the hippocampus.^[^ ^62^ ^]^ Currently, imaging neurons in the brain have relied on histology or immunohistochemistry, which is challenging to image large volumes of human brain tissue. Imaging cells using nonlinear label‐free methods have been reported earlier.^[^ ^42^ ^]^ Here, we demonstrate that with serial sectioning two‐photon microscopy, we can identify individual cells using lipofuscin‐dark‐spot structure, and provide reliable cell counting comparable to Nissl stain. This demonstrates the potential for a label‐free cell counting method. Although the validation and quantification of autofluorescence was conducted at sub‐micro resolution by a separate 2PM in this study, recent advancements in machine learning methods show promise as a means of segmenting low‐resolution images using prior knowledge from high‐resolution ones.^[^ ^63^, ^64^ ^]^

Lipofuscin has been for a long time considered an aging pigment. However, Morawski et al^[^ ^39^ ^]^ found the vulnerability of some neurons to accumulation of lipofuscin at the absence of perineuronal nets (PNN), an extracellular matrix structure that protects cells from oxidative stress. In addition, Brückner et al^[^ ^40^ ^]^ found an inverse correlation between PNN and neurofibrillary tangle. Therefore, lipofuscin could potentially serve as a biomarker in neurodegenerative diseases. Our method provides large‐scale quantitative analysis of lipofuscin distribution and can be studied together with myelin defects at the same region. We found increased lipofuscin area fraction with aging in Section 2.4, which could be one of the sources that contributed to the increased µ_*s*_ in aged brain tissue in Section 2.2. This potential correlation between optical properties and lipofuscin demonstrates our ability of studying multiple perspectives of aging process and coherently integrate them for better interpretation, which can be applied to neurodegeneration as well.

In summary, our method has provided multi‐contrast and multi‐resolution measurements of myelin content, vasculature, lipofuscin, and neuron density at large scale and high throughput in the human brain. This enables novel directions in the study of aging and neurodegenerative diseases, including demyelination, vascular dysfunction, and cellular modification. Particularly, with all structures measured simultaneously and co‐registered, our system allows the investigation of their spatial correlation at different stages during disease progression. Previous studies have observed neuron loss, myelin degradation, and vascular changes in cerebrovascular disease, Alzheimer's disease, and normal aging.^[^ ^55^, ^57^, ^65^, ^66^, ^67^, ^68^, ^69^ ^]^ However, the temporal order and the spatial relationships between these pathological features are still unclear. Our method, which provides volumetric reconstruction and quantitative analysis of large volumes of human brain tissue offers a powerful tool to further understand these brain disorders.

## 4 Experimental Section

### Sample

Three 4 × 4 × 2 cm^3^ brain samples were obtained from Massachusetts General Hospital Autopsy Suite. They were neurologically normal samples (PMI less than 24 h), and were used for brain structure imaging in Figures 1, Figure 2a–d,  3, and 6. One CTE stage I sample (26 years old male, Figure 4) and five normal control samples (64 to 88 years old, all male, PMI from 13.5 to 43.5 hours, Figures 3e–h and 5) were obtained from Boston University Alzheimer's Disease Research Center brain bank. Out of the five normal control samples, only three samples with PMI less than 24 hours were used in Figure 3e‐h. All five normal control samples were used in Figure 5f‐k. These samples were fixed by immersion in 10% formalin for at least two months, ^51^ washed in phosphate‐buffered saline for a month to remove residual fixation agents and then embedded in 4.5% agarose for tissue support.^[^ ^52^ ^]^ During embedding, the brain blocks were warmed to 65 °C to allow complete penetration of agarose into the deep sulcus. A 3D printed base plate was embedded onto the bottom of the agarose block and screwed to the bath container to mount the tissue block during the one‐week serial block‐face imaging (inset in Figure 1). The samples in Figures 1 and 2 were refractive‐index matched in 60% 2,29‐thiodiethanol (TDE).^[^ ^70^ ^]^ The index‐matching procedure was described in detail previously.^[^ ^70^, ^71^ ^]^ The other samples were immersed in DI water during imaging. The immersion liquid was changed every day to remove the debris from cutting that could degrade the image quality.

### PSOCT‐2PM System

The hybrid system consists of the PSOCT, 2PM, a custom‐built vibratome, XYZ motorized stages, and the control software for the whole system. The PSOCT microscope used a swept light source (AxsunTech) with 100 kHz swept rate, a central wavelength of 1310 nm, and a spectral full‐width half maximum of 110 nm, yielding an axial resolution of 5.6 µm in brain tissue (*n* = 1.4). At the interferometer, a polarization controller (Thorlabs Inc) and a polarization beam splitter (PBS) ensure maximum power input with horizontal polarization. A 50:50 beam splitter (BS) separates the light intensity equally onto the sample arm and reference arm. In the sample arm, an achromat quarter‐wave plate (QWP) is placed in front of the sample arm at 45 degrees to generate circular polarization on the sample. The scanning path consists of a pair of galvo mirrors (Thorlabs Inc), a 4x telescope, and a 4x objective (Olympus, UPLFLN4x, NA = 0.13), providing 5 µm lateral resolution and a 150 µm confocal parameter. In the reference arm, a linear polarizer (LP) is placed in front of the reference arm at 45 degrees to make the light intensity equal between horizontal and vertical polarization states. A retroreflector (RF) is placed at the end of the reference arm to back‐reflect the light. The back‐reflected light from the sample arm and the reference arm interferes when re‐combined in the interferometer and is split by two PBS into horizontal and vertical polarization states, which, through balanced detection, result in the co‐ and cross‐polarization measurement of the interference pattern. To acquire the spectrum in even *k*‐space, the k‐clock of the light source was fed into a high‐speed digitizer (ATS9350, AlazarTech) as a sampling clock. A Graphic Processing Unit (RTX4000, NVIDIA) was used to perform real‐time FFT, and the spatial‐domain data was trimmed to only save the first 1 mm depth. The postobjective power was measured to be 3.7 mW, achieving a 95 dB SNR in both polarization channels. For large samples, a 3 × 3 mm^2^ FOV was used with 3 µ*m* lateral step size and 10% overlap between tiles. For small samples and focusing on capillaries, a 1.5 × 1.5 mm^2^ FOV was used with 3 µm step size and 10% overlap.

The optical components in the sample arm generated a large system birefringence that caused substantial errors in the retardance and optic axis orientation measurements. To address this problem, we used a variable retarder (VR) in the sample arm, assuring the measurement to be pure tissue birefringence (Figure 2). The compensation process can be carried out in 3 steps:

1. We imaged a silver mirror and calculated the retardance. Assuming there was no system birefringence, the measured retardance should be 0. So, the none‐zero retardance we measured was the accumulated retardance from the optics.
2. We set the retardance of the variable retarder to be opposite of the value we calculated in Step 1, placed it before the galvo mirrors, and rotated it to minimize the signal from mirror in the cross‐polarization channel.
3. We then fine‐tuned the retardance and orientation of the variable retarder iteratively to minimize the cross‐polarization channel signal of sliver mirror.

The 2PM used a Ti:Sapphire mode‐locked laser with 80 MHz rep rate, 100fs pulse duration, and ≈3 W laser power at 820 nm. Right after the laser output, an attenuator made of a motorized half‐wave plate and a PBS was used to modulate the power input to the microscope system. Actual power on the sample was 128 mW. Following the attenuator, a 1.25x beam expander combined with the 4X telescope in the scanning path expand the beam size to fill the back‐aperture of the objective. The 2PM beam and PSOCT beam in the sample arm were combined using a dichroic mirror above the objective. Sharing the same objective with PSOCT, 2PM achieved a 2.4 µm optical lateral resolution and 48 µm axial resolution. For large samples, a 3 × 3 mm^2^ FOV was used with 2 µm lateral step size and 10% overlap between tiles. For small samples and focusing on capillaries, a 1.5 × 1.5 mm^2^ FOV was used with 1 µm step size and 10% overlap. The depth of focus of 2PM was overlapped with the first one‐third of PSOCT, assuring that they imaged the same region of the brain tissue while 2PM had the best contrast. For small samples and focusing on capillaries, 2PM was imaged at three different depths to cover the whole PSOCT depth of focus. Two detection channels were available, the short wavelength channel covered from 435 to 485 nm and the long wavelength channel covered from 500 to 700 nm to capture lipofuscin autofluorescence.

Two high‐performance computers controlled the PSOCT and 2P system separately, which were synchronized using frame start and stop triggers. For the 2PM system, we used the ScanImage (Vidirio Inc) to control the 2PM acquisition. For the serial sectioning PSOCT system, we wrote custom software in LabVIEW to automate the PSOCT acquisition, XYZ stages, and vibratome slicing. Each PSOCT c‐scan generates 7.6 GB of spectral domain data, which is challenging to save within 16 s of acquisition time. The authors used a GPU (Nvdia RTX4000) for performing FFT on the fly, and with depth trimming we reduced the saved data size to about 2.5GB. Still, for a 4 × 4 × 2 cm^3^ brain sample the PSOCT system will generate 60–70 TB data, which were stored in the remote Boston University Shared Computing Cluster (BU SCC) through 10Gb/s Ethernet connection. In case of network breakdown, we have 20 TB RAID10 local disk space for temporary storage. The data was stored at SCC for postprocessing, including the distortion correction and stitching, which further reduced the data size to about 30 TB at original resolution, and 5–7GB volume after 10 × 10 × 10 pixel down‐sampling.

### System Characterization

The authors characterized the lateral and axial resolution of both microscopes (Figure S1, Supporting Information). Using 1 µm fluorescent beads, the lateral and axial resolution of 2PM was measured to be 2.4 and 48 µm, respectively (a, b). The lateral resolution of PSOCT was 5 µm, where the modulation transfer function showed 50% contrast between the line‐pair (c). Using the air‐glass interface and considering the brain tissue index of refraction (*n* = 1.4), the axial resolution of PSOCT was measured 5 µm (d). The confocal parameter was measured to be 150 µm using an intralipid phantom (Figure S2, Supporting Information).

In swept source OCT, the sensitivity drops along depth due to the limited spectral resolution of the swept light source, yielding a sensitivity roll‐off. Figure S1e, Supporting Information shows the sensitivity roll‐off measurement at different reference mirror positions. We found the sensitivity remained unchanged over the first 1 mm depth. The purple horizontal line represented the fitting of the peak intensities using Equation 2.^[^ ^72^ ^]^ As the brain signals were captured within the first 1 mm, the small sensitivity roll‐off relieved us from performing roll‐off compensation during post‐processing.

The authors performed a thorough characterization of the polarization measurements in PSOCT before and after compensating the system birefringence. To obtain the polarization extinction ratio (PER, Figure 2a), we used a microscope glass slide as sample and rotate the QWP in the sample arm to get maximum and minimal signals from the cross and co‐polarization channels. PER was then calculated by ratio of the maximum and the minimum intensity signal from the same channel. To characterize the retardance and orientation measurements (Figure 2b,c), a second variable retarder was used as the sample and imaged the bottom surface of the retarder. The voltage of the retarder was adjusted to change the retardance and rotated the retarder to change the optic axis. It was then compared the PSOCT‐measured retardance and orientation with the true retardance provided by the manufacturer and the actual orientation of the retarder.

### Image Acquisition and Postprocessing—Data acquisition and Real‐Time Processing

Block‐face imaging and serial sectioning were performed on three human brain blocks. For the sample without index matching, PSOCT imaged 150 µm deep into the tissue with 5 µm resolution, while 2PM acquired a single plane integrating a depth range of 48 µm (axial resolution). Then a 150 µm thick slice was cut off by the vibratome. During serial sectioning, it was also cut 30 µm thick slices for Nissl stain. For the sample with index matching, PSOCT could effectively image 450 µm deep into the tissue. This was achieved by imaging the sample at two different focal depths, separated by 225 µm. Two 2PM images were taken at the same time. Then a 450 µm thick slice was cut by the vibratome.

### Image Acquisition and Postprocessing—Distortion Mitigation and Correction

The optical distortions include geometric distortions in the XY plane (grid distortion), XZ and YZ plane for PSOCT (field curvature distortion), and intensity distortion in the XY plane (shading distortion), axial dimension (depth attenuation for PSOCT). Since the two geometric distortions depend purely on the optical system, precalibration could be applied to correct them. Briefly, a grid target (Thorlabs Inc) parallel to the table surface was imaged before the experiment. The back‐reflection signal off the top surface shared the same field curvature as any well‐cut sample. A 2D look‐up table of the curvature was obtained from the grid target and applied to the brain images to flatten the signal surface. Next, the average intensity projection (AIP) of C‐scan images of the grid target along depth provided the warped image of the grid that was used in grid distortion correction. An ideal grid pattern was generated in MATLAB with the same pixel spacing and orientation as the captured image. Using the UnwarpJ plugin of ImageJ, the warped grid pattern was registered to the nondistorted pattern and a transformation matrix was generated that could be used to unwarp the sample images. A similar procedure was applied to correct the 2PM images. For the shading distortion, it was employed the BaSiC shading correction algorithm,^[^ ^73^ ^]^ using the ImageJ plug‐in. For the depth attenuation, a high‐pass filter was used for each XY frame of OCT volume to remove intensity variation, and normalize the mean intensity of each XY frame to smooth the axial intensity variation.

Cutting distortions are mitigated by the serial sectioning scheme. During sectioning, the slice being cut and the tissue beneath the blade can experience tissue loss and distortion. However, a rule of thumb is that the tissue 50um below the surface, for most cuts, will be distortion‐free. Considering this, the focus ≈50um below the surface was placed. For PSOCT, the depth of focus is between 50–200um below the surface. For 2PM, the depth of focus was 50—98 um below the surface. In PSOCT, a total of 1 mm depth was collected, including some space above the tissue, and all the way down to 600–700um below the surface. During volume reconstruction, it is only used the depth range that is in focus, i.e., between 50 and 200um below the tissue surface. This way, it could confidently mitigate the cutting distortion. For the continuity in the axial direction, since the XYZ stage had ≈10 um precision, which was only slightly larger than the resolution, overlapping was not necessary during volume reconstruction.

### Image Acquisition and Postprocessing—Stitching and Volume Reconstruction

The hybrid system generated two sets of data, the speckle‐noise contaminated PSOCT images and the autofluorescence images. As the two modalities were acquired synchronously, the same coordinates could be used to stitch both images. Because 2PM images had better SNR, 2PM images were stitched to get stitching coordinates and then used them to stitch PSOCT images. We used the stitching plugin of ImageJ to find the optimal stitching coordinates for the 2PM images. The authors selected three planes separated at different depths of the volume, merging them into a RGB format and ran the stitching plugin to find the global optimal coordinates for stitching and applied them for all the slices, which were then stacked to reconstruct the volume.

### Image Acquisition and Postprocessing—Scattering and Birefringence Fitting

Extraction of scattering coefficient,^[^ ^71^, ^74^ ^]^ back‐scattering coefficient, and birefringence^[^ ^30^ ^]^ followed previously reported methods, by fitting the depth profiles of PSOCT intensity and retardance.

### Image Acquisition and Postprocessing—Registration between PSOCT and 2PM Images

As PSOCT and 2PM were acquired simultaneously, and all distortions have been corrected in previous steps, registering these two modalities only need to scale PSOCT and 2PM to the same pixel size and then find out how many pixels were shifted between the two FOVs in X and Y dimensions. The authors determined this shift by finding the maximum overlap between the two images on the same vessel. The scaling factor from PSOCT to 2PM was 1.5. The shift from PSOCT to 2PM was −50 pixels in X dimension and 113 pixels in Y dimension.

### Image Acquisition and Postprocessing—Histology Process and Imaging

The Nissl staining protocol was described previously.^[^ ^75^ ^]^ We used a microscope slice scanner (Olympus, VS200) for imaging the Nissl‐stained slices. A 20x objective was used, with a lateral resolution of 0.5 µm. The commercial software VS200 performed intensity correction and stitching automatically.

### Image Acquisition and Postprocessing—Bruker 2PM Imaging

After imaging under our hybrid system, it took the sample to a Bruker 2PM for high‐resolution imaging. A 20x water objective was used, yielding a lateral resolution of 0.5 µm and axial resolution of 1.3 µm. The authors used 820 nm excitation wavelength, the same as in the hybrid system. The emission filter was 500–550 nm, revealing both the lipofuscin and the dark spots as shown in the hybrid system. Z‐stacking was carried out in 10 µm steps and a total of 30 µm depth was imaged. Two‐dimensional autofluorescence images were generated from the Z‐stack. Lipofuscin map was obtained by the maximal intensity projection of Z‐stack and the dark spot map was obtained by the minimal intensity project.

### Image Acquisition and Postprocessing—Fluorescence Lifetime Imaging

Fluorescence lifetime image was also carried out at the Bruker 2PM system. The image settings were same as Brucker 2PM imaging. Fitting of a lifetime was carried out using the commercial software SPCImage.

### Image Acquisition and Postprocessing—Lipofuscin Quencher

We used the TrueBlack (Biotium Inc) lipofuscin quencher, the processing protocol can be found on the website: https://biotium.com/product/trueblack‐lipofuscin‐autofluorescence‐quencher/. We applied the quencher to a 30 um thick fixed brain slice and imaged the autofluorescence under Bruker 2PM before and after quenching.

### Segmentation of Lipofuscin and Quantitative Metrics

Segmentation of lipofuscin was based on high‐pass filtering of the image and adaptive thresholding afterward. Specifically, the process was carried out in the following steps:

- Low‐pass filtering was applied on the 2PM long wavelength channel using the Gaussian smoothing function of ImageJ. It was used a Sigma (radius) value of 50, which corresponded to 100 µm.
- The original image on the 2PM long wavelength channel was divided by the filtered image in (a). This way it was removed the low‐frequency fluctuation in the background.
- The image was binarized using the Threshold function of ImageJ and Huang method on the image in (b). The lower bound 1.22 was used, which gave the best discrimination between lipofuscin and background.

The bright tissue boundary could be mistaken as lipofuscin in this segmentation routine, but they only contribute a small portion of the total area, and hence the contribution was negligible in the results. From the lipofuscin segmentation, quantitative metrics were calculated by employing a sliding window method. The authors used a 200 × 200 µm window size (100 × 100 pixel) and 100 µm step size, within each window the total pixels of lipofuscin *P*_1_were summed and number of isolated lipofuscin granules N are counted. The area fraction is calculated by *P*_1_/100/100, the number density is calculated by N/(0.2×0.2mm^2^), and the mean radius is calculated by $\sqrt{\frac{P_{1}}{\pi N}*4\;{\mathrm{um}}^{2}}$. Linear regression analysis of the lipofuscin metrics with age was conducted in MATLAB using least‐square fitting function.

## Conflict of Interest

The authors declare no conflict of interest.

## Supporting information

Supporting Information

Supplemental Video 1

Supplemental Video 2

## Data Availability

The data that support the findings of this study are available from the corresponding author upon reasonable request.
