# Supplementary material for: Multi‐Scale Label‐Free Human Brain Imaging with Integrated Serial Sectioning Polarization Sensitive Optical Coherence Tomography and Two‐Photon Microscopy
Source: Adv Sci (Weinh). 2023 Oct 26;10(35):2303381. doi: 10.1002/advs.202303381 (PMC10724383; doi:10.1002/advs.202303381)
Supplement: Supplementary file 1 — Supporting Information [file ADVS-10-2303381-s001.pdf]

ADVANCED SCIENCE
Open Access

Supporting Information

for *Adv. Sci.*, DOI 10.1002/advs.202303381

Multi-Scale Label-Free Human Brain Imaging with Integrated Serial Sectioning Polarization Sensitive Optical Coherence Tomography and Two-Photon Microscopy

*Shuaibin Chang, Jiarui Yang, Anna Novoseltseva, Ayman Abdelhakeem, Mackenzie Hyman, Xinlei Fu, Chenglin Li, Shih-Chi Chen, Jean C. Augustinack, Caroline Magnain, Bruce Fischl, Ann C. Mckee, David A. Boas, Ichun Anderson Chen and Hui Wang**

Supplementary Figures

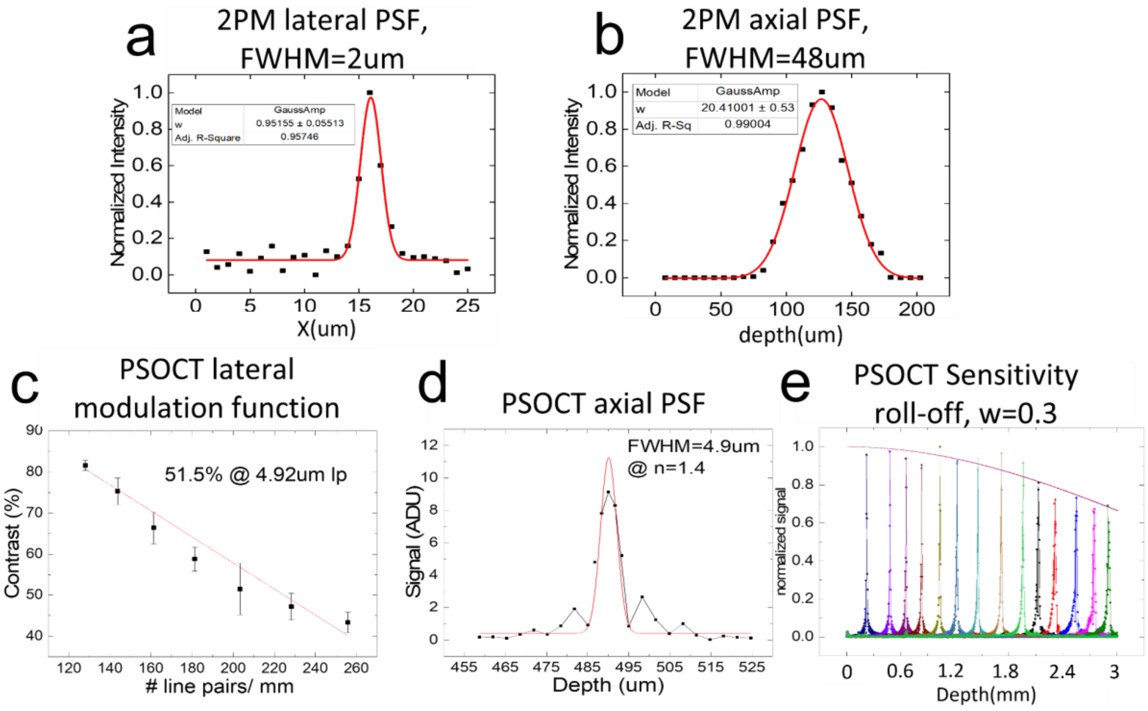

Supplementary Figure 1. Lateral and axial resolution measurement of the PSOCT-2PM system. (a) lateral resolution of 2PM measured to be 2 μm using 1 μm diameter fluorescent beads. (b) axial PSF of 2PM measured to be 48 μm. (c) Lateral modulation function of PSOCT system using airforce target shows 50% contrast at 5 μm line pair. Red line shows linear approximation of theoretical values. (d) Axial PSF of PSOCT measured to be 5 μm using glass slide surface. (e) Sensitivity roll-off of the PSOCT signal over depth.

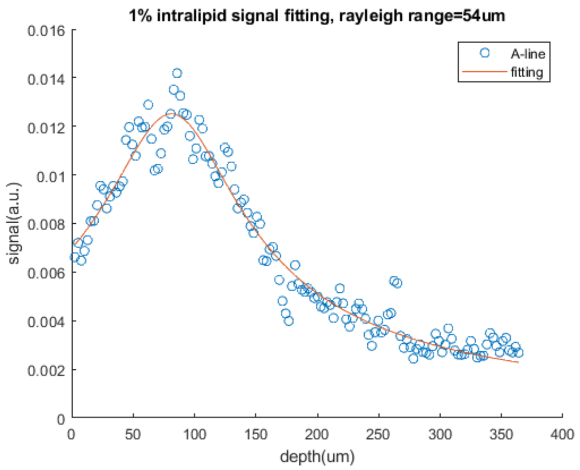

Supplementary Figure 2. Four parameter optical fitting of 1% volume concentration intralipid solution. Rayleigh range was estimated to be 54 μm, which corresponds to 108 μm of confocal parameter. With higher scattering, effective Rayleigh range would increase[71], we found in brain tissue the confocal parameter is about 150 μm.

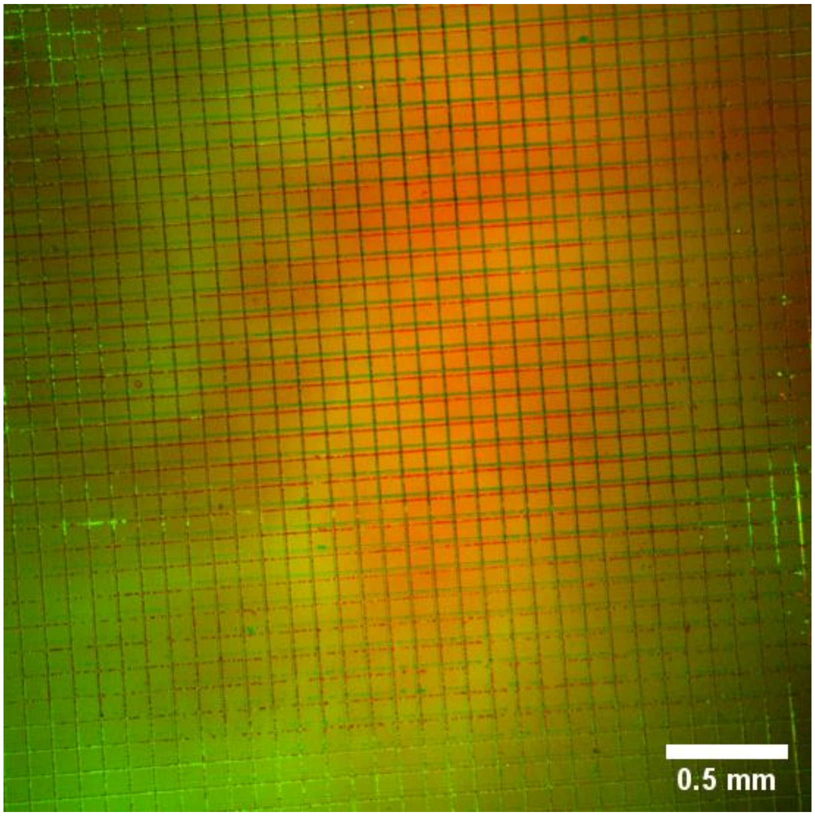

Supplementary Figure 3. Grid distortion of the PSOCT system 3mm FOV with air and water immersion. Red: air. Green: water immersion. Note there are linear shifting of the red and green grid, and with minor non-linear distortion between the two immersions. The linear shifting does not affect image quality, it's only the non-linear shifting that matters, which is very small.

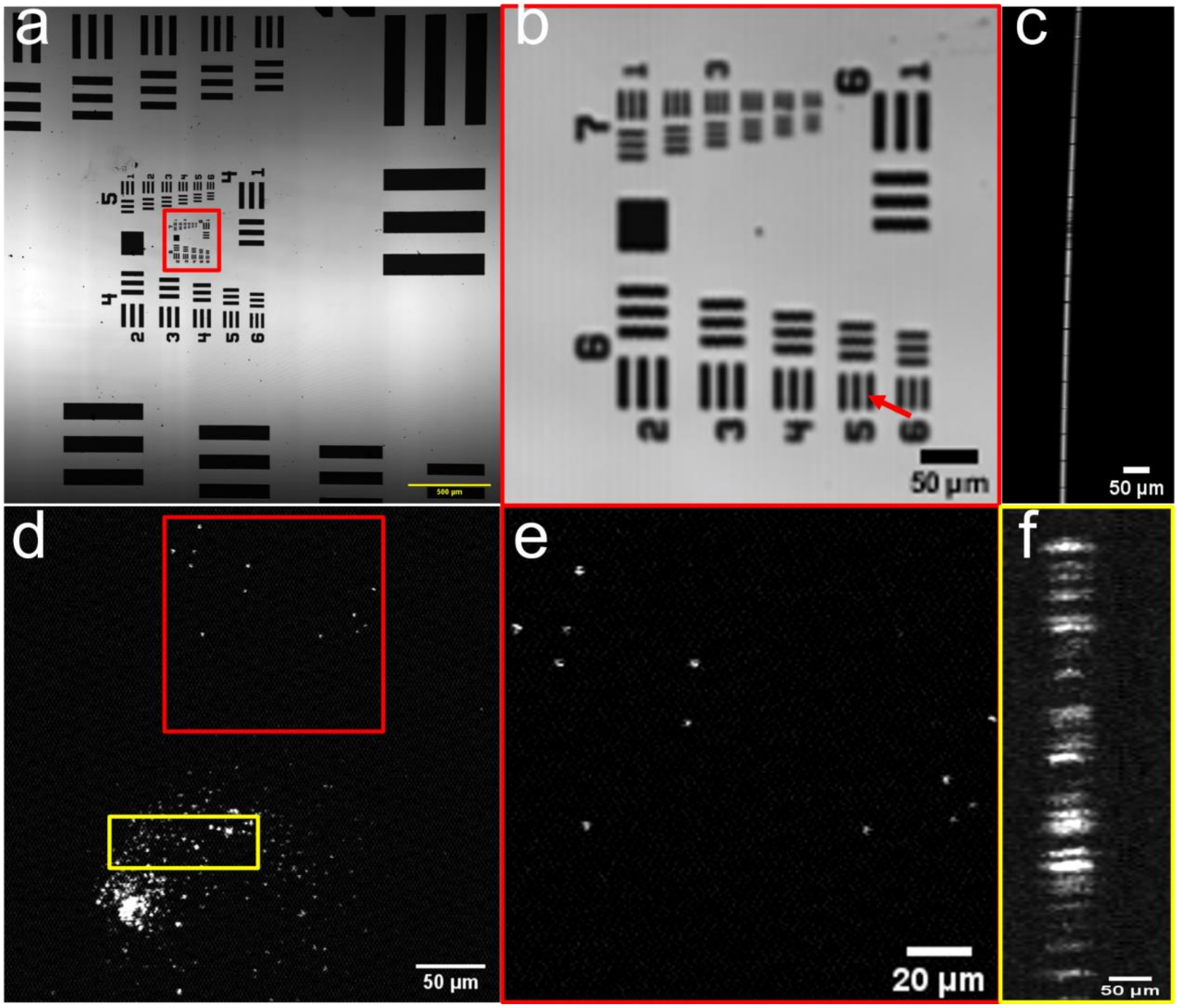

Supplementary Figure 4. Original images of resolution characterization. (a-b) PSOCT resolution measurement. (a) air-force resolution target of the whole FOV. (b) zoom into the group 6 of airforce target, the line width of Group 6 line 5 (red arrow) is 4.92um. (c) B-line of a grid target, the air-glass interface was shown as the bright line. (d-f) 2PM resolution measurement using 1um fluorescent beads. (e) zoom in of the beads in red ROI in (d). (f) XZ plane of beads in yellow ROI of (d).

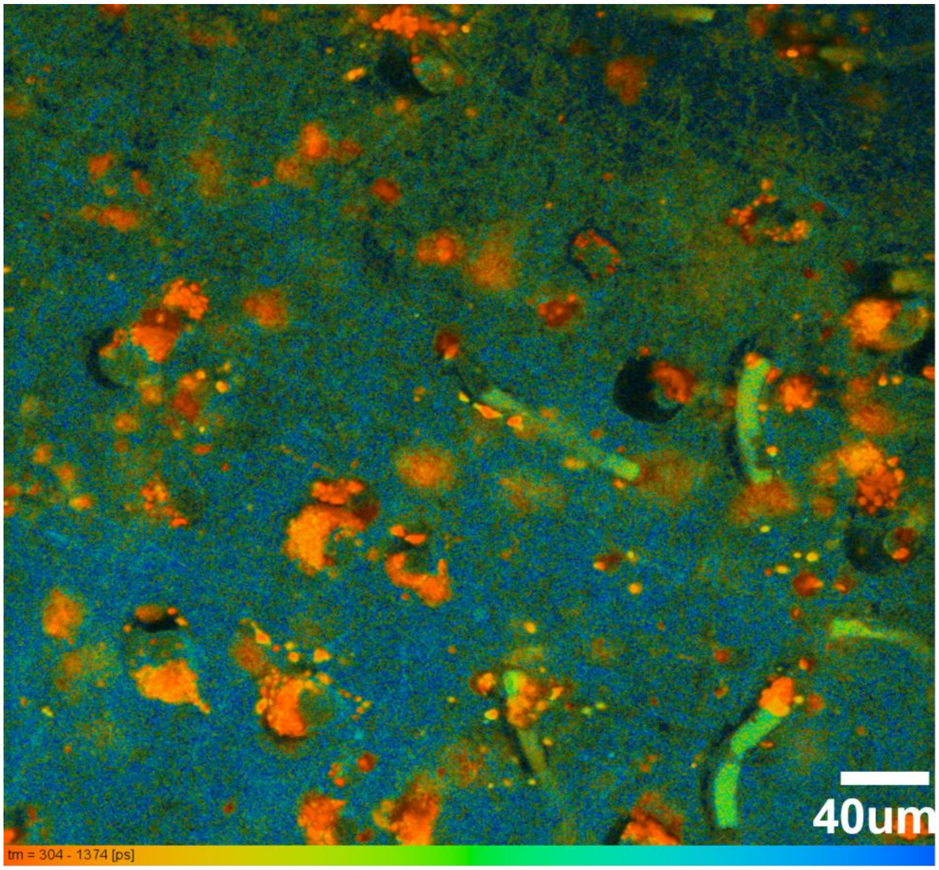

Supplementary Figure 5. Fluorescence lifetime imaging of fixed human brain tissue. The autoflurescent structures alongside with the dark spots have about 300-400ps lifetime.

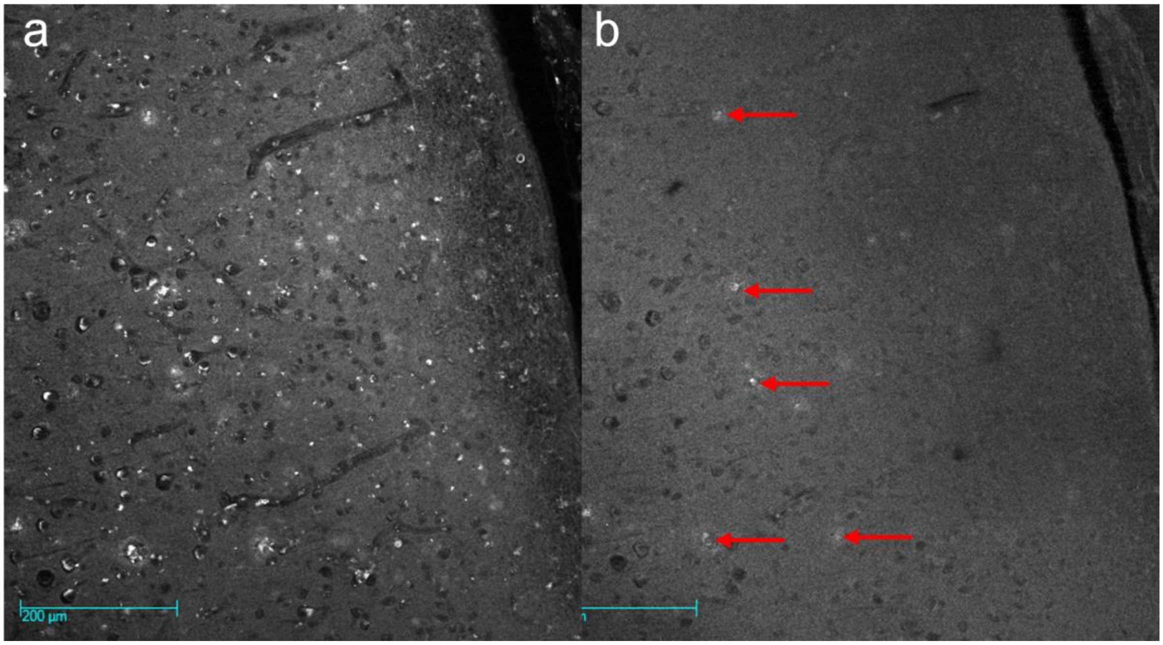

Supplementary Figure 6. Autolfuorescence 2PM image of AD brain slice before (a) and after (b) lipofuscin quencher. The bright particles before quenching are gone in b. Red arrows highlight some structures that didn't get quenched, which are possibly amyloid beta plaques.

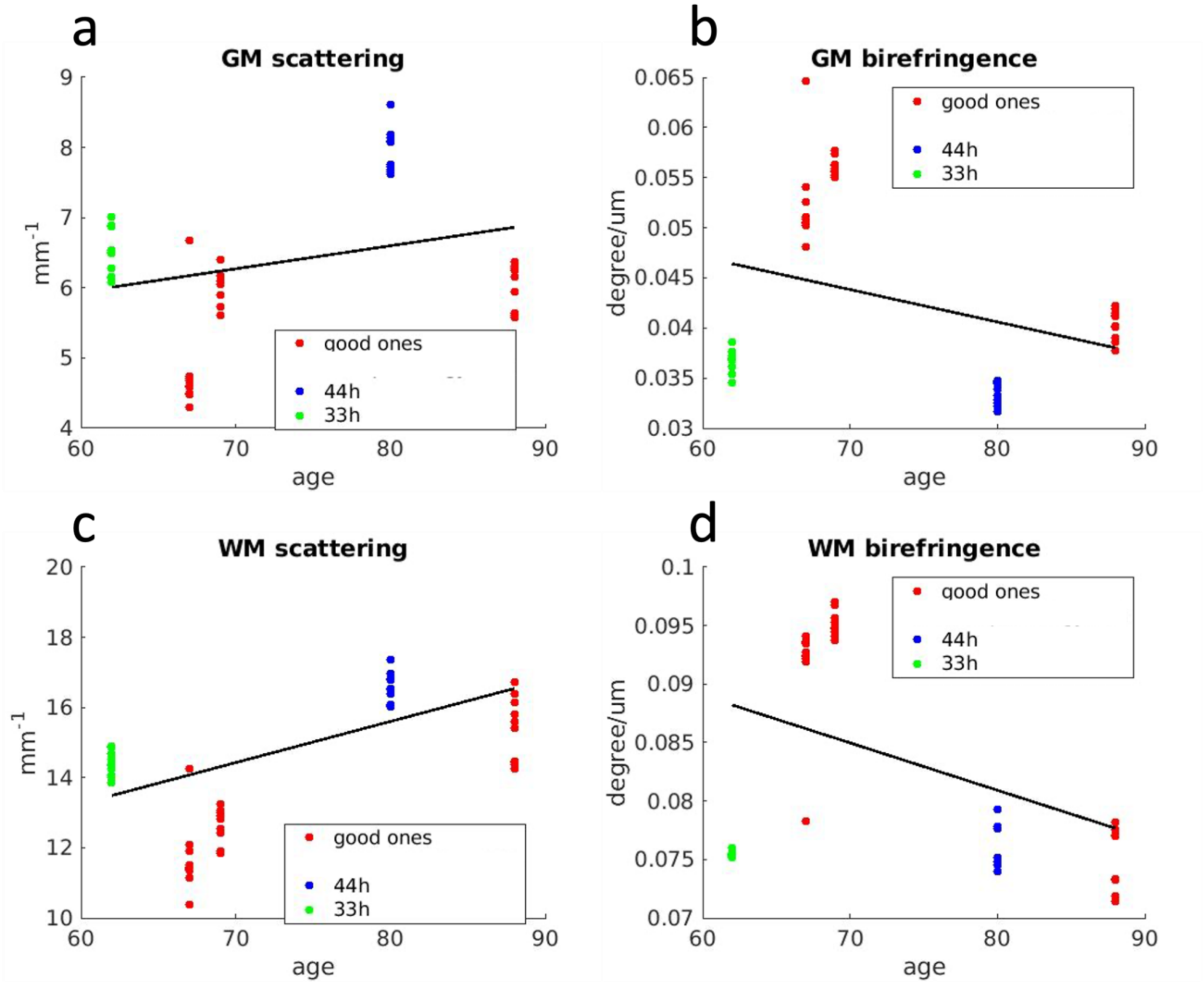

Supplementary Figure 7. Quantitative comparison of μ_s and retardance for all five normal control samples, the three samples with less than 24 hours of PMI are in red, the other two samples have 33 and 44 hours of PMI, respectively. Comparing to the samples with short PMI, the long PMI samples have increased scattering and reduced birefringence.
